# Characterization of the Early CNS Stress Biomarkers and Profiles Associated with Neuropsychiatric Diseases

**DOI:** 10.2174/138920212802510448

**Published:** 2012-09

**Authors:** XR Lowe, AJ Wyrobek

**Affiliations:** 1 Life Sciences Division, Lawrence Berkeley National Laboratory, Berkeley, CA 94720, USA; 2 Department of Psychiatry, Kaiser Permanente^®^ Medical Center, Hayward, CA 94587, USA

**Keywords:** Early CNS stress biomarker, Interferon, Radiation, Low-dose, Ketamine, Microarray, Neuropsychiatric diseases, RNA *in situ* hybridization, Tnnt1.

## Abstract

Neuropsychiatric disorders (including dementia) have high personal, family, and social costs. Although many neuropsychiatric disorders share common patterns of symptoms and treatments, there are no validated biomarkers that define the underlying molecular mechanisms in the central nervous system (CNS). We hypothesize that there are early and common molecular changes in the CNS that will serve as sensitive indicators of CNS molecular stress and that will be predictive of neuropathological changes resulted in increasing the risk for neuropsychiatric diseases. Using the rodent model, we showed that systemic exposure to three diverse CNS stressors with different mechanisms of action (ketamine, low-dose and high-dose ionizing radiation, interferon-α) induced the expression of troponin T1 (Tnnt 1) within hours in adult mouse brain tissue. Tnnt 1 expression was induced in neuronal (not glial) cells, the hippocampal zone of neurogenesis, cerebral cortex, amygdale, and choroid plexus, which are important CNS locations in behavior and mental health. We also identified nine neural signaling pathways that showed a high degree of concordance in their transcriptional response in mouse brain tissue for hours after low-dose irradiation, in the aging human brain (unirradiated), and in brain tissue from patients with Alzheimer’s disease. Our studies provide new molecular information on shared mechanisms and expression profiles of diverse neuropsychiatric disorders. This knowledge will be fundamental for developing molecular signatures of early CNS stress biomarker for early diagnosis and treatment of neuropsychiatric diseases.

## INTRODUCTION

The prevalence of major neuropsychiatric disorders such as schizophrenia, bipolar disorder, major depressive disorders and dementia is high, with little insight into underlying causes and molecular defects. Severe forms of depression affect 2-5% of the U.S. population, and up to 20% suffer from milder forms of this illness. Approximately 1%–2% of the populations are afflicted by bipolar disorder [1] and ~ 1% suffers from schizophrenia. The Alzheimer’s Association estimates that one in eight, or 13 percent of people over age 65 have Alzheimer’s disease. The prevalence of Alzheimer’s disease (AD) and other dementias will continue to increase with the rapid growth of our older population [2, 3]. The major neuropsychiatric disorders are thought to arise from complex genetic and environmental interactive factors, and a common clinical observation is that they are generally preceded by periods of biological, psychological and/or social stress. A sensitive preclinical biomarker of CNS stress would be an important asset for practitioners to identify early risk factors or early onset of neuropsychiatric disease when intervention is known to be most helpful. A sensitive biomarker of CNS stress might also help to prevent tragic outcomes and to avoid persistent mental impairments. In most other organ systems, there have been significant advances in biomarker development for complex disorders (e.g., serum glucose and IgA1C to monitor diabetes, or serum troponin to diagnose acute myocardial infarction). Unfortunately, to date, there are no validated biomarkers for CNS stress or risks for neuropsychiatric diseases [1]. Thus, a successful biomarker(s) of CNS stress will be a major advance in the field.

The treatment of major neuropsychiatric diseases was revolutionized by the introduction of several classes of medications (such as lithium, haloperidol, tricyclic antidepressants, atypical anti-psychotics, Selective Serotonin Reuptake Inhibitor (SSRI) anti-depressants, and Memantine for dementia). Yet, our progress in understanding the genetic and neurobiological mechanisms underlying neuropsychiatric diseases has been disappointing [1]. At best, only about 65 % of patients with mood disorders respond adequately to available drugs [1]. Electric convulsive treatment (ECT) is still the most effect treatment for depression, mania and catatonic psychosis. As for dementia, with an estimated worldwide prevalence of AD of ~30 million and a quadrupling of numbers expected by 2050, a definite early diagnosis remains uncertain and there currently is no effective treatment that delays the onset or slows the progression of AD [4]. It is imperative that we identify biomarkers of CNS stress and understand the mechanisms underlying CNS stress responses and their associations with the onset and evolution of neuropsychiatric diseases. 

The mechanisms by which nongenetic and environmental factors such as bio-psycho-social stress influence the risk of neuropsychiatric disorders remain uncertain and inter-individual variation in response is typically large and similar treatments can be effective for apparently different disorders. For example, interferon-α (IFN-α) treatment, which has been used for the treatment of certain viral illnesses, cancers, chronic hepatitis C, can induce a wide range of psychiatric symptoms. More than 30% of patients treated by IFN-α presented various psychiatric disorders including depression, anxiety, intense and fluctuating personality disorders, manic or psychotic symptoms, and suicidal tendencies [5]. In addition, some neuropsychiatric disorders have symptom patterns and treatment that cross disciplinary boundaries. Lichtenstein *et al.* (2009)[6] reported that schizophrenia and bipolar disorder partly share a common genetic cause in a population-based study. In addition, most medications developed as anticonvulsants have beneficial effects on bipolar disorder [7]. Also, the same disorder can be treated by completely different approaches. For instance, depression can be effectively treated by monoamines (serotonin, norepinephrein and dopamine), psychotherapy or ECT regardless of its functional mechanism. Based on these clinical observations, we hypothesize that there are common early stress response mechanisms (a gate keeper) in the CNS after exposures to diverse environmental stress/insults Fig. (**1**). In support of this hypothesis, we have performed a comparative study of the early CNS stress response in a rodent model after exposure to three agents including ketamine, ionizing radiation and IFN-α. As summarized below, these three agents have different effects on the CNS.

### Ketamine

The highly complex glutamatergic system has been associated with a variety of neuropsychiatric disorders, e.g., schizophrenia and bipolar disorder [8, 9], Alzheimer's Disease [10] and seizure disorder [11]. Clinical treatment with a non-competitive glutamate N-methyl- D-aspartic acid (NMDA) receptor antagonists such as ketamine, induce a broad range of cognitive adverse effects including deficits in working memory, verbal fluency, vigilance tasks, and symptoms that resemble various aspects of schizophrenia (dose-dependence). Yet, NMDA antagonists can both induce and prevent neurotoxicity associated with excitotoxicity [12]. Memantine, an NMDA-receptor antagonist, has been approved by the U.S. Food and Drug Administration (FDA) for the treatment of Alzheimer's disease [10]. Zarate *et al.* (2006)[13] reported robust and rapid antidepressant effects following a single intravenous dose of ketamine in humans. The paradoxical characteristics of the NMDA receptor blocking drugs, such as ketamine, make these drugs valuable in the study of the pathophysiology of neuropsychiatric illness and medical comorbidity [14].

### Ionizing Radiation

External-beam radiotherapy of the human brain has been associated with neurological damage and cognitive impairment, especially in children [15, 16]. Organic brain damage and accelerating CNS aging were reported long after exposure to < 1 Sv in the Chernobyl nuclear reactor accident [17]. The neurological deficits of high-dose radiation are progressively detrimental over time and are thought to be due to demyelination and neural loss [18] with associated neural and cognitive deficiencies [19]. Some of these cognitive defects have been observed as a consequence of impaired neurogenesis after exposure to ionizing radiation.

### IFN-α

Cognitive impairment and mood disorders are well recognized but poorly understood sequelae of IFN-α therapy [5]. Wang *et al.* (2008)[20] provided evidence that mouse IFN-α, circulating systemically, can enter the CNS and act in the brain locally through signal transducers and activators of transcription (STAT1). Orsal *et al.*, (2008)[21] reported that an increase of depression-like behavior in mice after systemic IFN-α exposure. 

For the current study, we utilized genome-wide expression profiling, RNA *in situ* hybridization techniques and fluorescence immunohistostaining Fig. (**2**) to search for candidate common early CNS stress response markers using mice treated with each of three different CNS stressors: ketamine, ionizing radiation and IFN-α that may produce symptoms that resemble various aspects of neuropsychiatric diseases and mimic the pathophysiologic conditions of diseases in the brain.

### Gene Expression Profiling in Mouse Brain After Ketamine, Ionizing Radiation and IFN-α Treatment

We utilized genome-wide expression profiling to characterize the response of 8-10 week-old B6C3F1 male mice brain exposed to ketamine (by a single intraperitoneal (i.p.) injection at 80 mg/kg), ionizing radiation (whole body γ radiation at 0, 10 cGy and 200 cGy, a ^137^Cs source with a dose rate of 0.64 Gy min^-1^), and to IFN-α (by a single i.p. injection at 1 x 10^5^ IU/kg ). RNA was isolated from the mouse brains tissues at various early times post treatment and hybridized to Affymetrix microarrays (MGU-74 av2 GeneChips®) Fig. (**2**). Several bioinformatics tools were used: GeneOntology (GO) enrichment analysis, Ingenuity Pathways Analysis (IPA 5.0), and L2L microarray analysis tool. GO analyses were performed using GO tree machine [22] to generate biological processes, molecular function and cellular component categories that were differentially associated with the ketamine-induced modulated gene set. GO categories were filtered based on significance of over-representation of ‘‘hits’’ by using a selected threshold for P values of hypergeometric distribution (P ≤ 0.001). The IPA knowledge base includes updated literature information on molecular networks and biological processes and an extensive library of well-characterized signaling and metabolic pathways to understand the transcriptional networks, phosphorylation cascades and protein-protein interactions. Modulated gene sets were also analyzed in IPA 5.0 for pathway enrichments with respect to reference chip MGU74Av2 to rank the top statistically significantly over-represented canonical signaling and metabolic pathways and to determine whether there was significant up- or down-regulation. The Fisher’s exact test was applied to examine the statistical over-representation of the pathways, using a threshold P value of ≤0.05. Differentially modulated genes were also overlaid onto the IPA knowledgebase interactome to identify networks that were significantly enriched. Generated networks were arranged according to IPA score (the higher the score, the lower the probability of finding focus genes in a given network by random chance). We used the L2L microarray analysis tool [ver 2007.1[23]] to identify other data sets that shared significant pathway enrichment with our radiation-induced gene set. L2L microarray analysis tool allowed us to conduct an unbiased search.

We hypothesize that there are common early stress response mechanisms (gate keepers) in the CNS that are induced after exposures to a broad variety of environmental stress/insults, and that these mechanisms are associated with increased risks for a broad range of neuropsychiatric diseases.

#### Ketamine Treatment

Approximately 50 genes were differentially expressed in ketamine-treated mouse brains compared with control mice that received i.p. injection with distilled water, (Table **S1**). Gene Ontology (GO) analyses showed that ketamine exposure of brain tissue induced significant biological processes, molecular functions and cellular components that were distinctly different from those treated with distilled water. Fig. (**3**) showed that ketamine-induced modulated genes were preferentially associated with metabolism, cell organization and biogenesis, intracellular signaling cascade and transcription in biological process analysis. The ketamine-induced signatures were significantly associated with protein and nucleotide binding in molecular function analysis Fig. (**S1**), and with the nucleus, cellular, membrane and cytoplasm component in cellular component analysis Fig. (**S2**). These GO analysis findings indicate that ketamine-induced damage responses in the brain are complex and functionally diverse and are significantly different from those of the controls. Troponin T1 (Tnnt1) gene showed consistent elevation (2- to 4-fold) across of the group of ketamine-treated mice. Tnnt1 nucleotides (GenBank accession number: AV213431 and AJ131711) showed ~ 4-fold increases for AV213431 (p <0.001) and ~ 2-fold increases for AJ131711(p< 0.03). Ingenuity Pathways Analysis (IPA) and network analysis were applied to the differentially expressed transcriptome profiles induced by ketamine exposure. Fig. (**4**) revealed that Tnnt 1 (oval) is associated with calcium canonical pathway (rectangle) and is regulated by FoxO1 (circle). Calcium metabolism plays a significant role in neuropsychiatric diseases in the CNS [24]. Fox O1 is a gene involved in multiple metabolic pathways including glycolysis, lipogenic and sterol synthentic pathways [25, 26], and central energy homeostasis.

#### Ionizing Radiation Exposure

Analyses of transcriptome profiles of mouse brain tissue 4 hours after whole body irradiation showed that low-dose exposures (10 cGy) induced genes not affected by high-dose radiation (200 cGy) and that low-dose genes were associated with unique pathways and functions. The low-dose response had two major components: pathways that are consistently seen across tissues and pathways that were specific for brain tissue. Low-dose genes clustered into a saturated network (P < 10^-53^) containing mostly down-regulated genes involving ion channels, long term potentiation and depression, vascular damage, and other functions [27, 28]. 

#### IFN-α Treatment

Analyses of transcriptional profiles of mouse brain tissue one hour post IFN-α treatment showed no significant differentially expression between the IFN-α treated and distilled water treated mice (Table **S1**). 

### Common Induced Expression of Tnnt 1 in Mouse Brain After Exposure to Three CNS Stressors

Troponin complex (especially Troponin I) has been used routinely in emergency rooms for diagnosis and monitoring acute myocardial stress and infarction [29, 30]. We hypothesize that the expression of Tnnt 1, a gene associated with calcium homeostasis, may be a common and early molecular stress-response in CNS. Therefore, Tnnt1 gene was chosen for further investigation of regional and cellular expression patterns using RNA *in situ* hybridization in the adult mouse brain after systemic exposure to ketamine, ionizing radiation and interferon-α, all of which have been associated with wide ranges of neuropsychiatric complications. 

Adult B6C3F1 male mice were treated with either ketamine (a single i.p. injection at 80 mg/kg), whole body gamma-radiation (0, 10 cGy or 200 cGy) or human IFN-α (single i.p. injection at 1 x 10^5^ IU/kg). Brain issues were isolated at 30 min post ketamine treatment and at 4 hours post IFN-α and irradiation treatment Fig. (**2**). Patterns of Tnnt 1 transcript expression were compared in various CNS regions after ketamine, radiation and IFN-α treatments. The control mice showed unique regional expression patterns for the Tnnt 1 gene (Table **1**): i.e., positive but weakly expression in the choroid plexus epithelium cells, ependymal lining of ventricles, and in the Ammon’s horn of hippocampus region and no detectable expression (negative) in pyramidal neurons and other regions. Tnnt 1 was induced in Purkinje cells of cerebellum after ionizing radiation and ketamine treatment; but not after IFN-α treatment. Tnnt 1 expression was consistently induced in pyramidal neurons of cerebral cortex, amygdale and hippocampal zone of neurogenesis after all three treatment regimens including 10 cGy of ionizing radiation (Table **1**). Tnnt 1 was consistently enhanced in the choroid plexus after all three exposures (Table **1**). Since the choroid plexus produces the majority of cerebral-spinal fluid (CSF), in the future, one might assess the status of Tnnt 1 modulation through CSF sampling.

IPA network analyses were applied to differentially expressed genes in mouse brain after in vivo ketamine exposure. The two top networks were merged to create the gene interaction network that involved Tnnt 1 (oval), which is regulated by Fox O1 (circle) and involved in the Calcium signaling pathway (rectangle). Dark gray color: up-regulated genes. Light gray color: down-regulated genes. White color: genes that were not differentially expressed after ketamine treatment.

Ketamine-induced Tnnt1 RNA expression was validated and characterized using fluorescence immunohistostaining in paraffin embedded brain tissue sections Fig. (**5**). We found that protein expression of Tnnt 1 was induced in mice treated with ketamine in the Purkinje cells, pyramidal neurons and in hippocampus region as shown in Fig. (**5A**). The patterns of protein expression of Tnnt 1 in CNS were consistent with those of RNA expression of Tnnt 1 Fig. (**5B**) [31]. The high concordance between the patterns of RNA and protein expression for Tnnt 1 in CNS Fig. (**5**)[32] indicating that Tnnt 1 was induced by ketamine at both RNA and protein levels.

Our studies allow us to compare the effects of treatment at the level of RNA expression (by microarray and RNA *in situ* hybridization) and the level of protein expression (by fluorescence immunohistostaining). There were some notable discrepancies among these endpoints in tissues. This may be due to the different animals, different sections of brain tissues and different sampling times were used. Microarray analysis did not show the induced-expression of Tnnt 1 after 4 hours irradiation treatment whereas we found increased RNA expression using RNA *in situ* hybridization. This discrepancy between microarray and RNA *in-situ* hybridization endpoints may be related to a difference in sampling site used for the microarray analysis where the entire coronal section was used [27] and for RNA *in* *situ* hybridization where the specific regions were used [32] Fig. (**2**). For IFN-α treatment, the disagreement in results between the microarray and RNA *in situ* hybridization may be due to the different sampling time, where one hour post-treatment was used in microarray and four hour post treatment was used in RNA *in situ* hybridization analysis. In addition, the discrepancies may also arise from the less sensitivity of microarray assay when compared to RNA *in situ* hybridization assay, as the evidence that Tnnt 1 expression that was about 4 fold increased detected by microarray while about 20 fold increased in Purkinje cells of mice treated by Ketamine (Table **1**).

### The Significance of Commonly Induced Tnnt 1 Expression in CNS Shortly After Exposure to Three CNS Stressors

Troponin T is the subunit of troponin complex and interacts with tropomyosin, troponin C, troponin I and F-actin; and troponin T1 (Tnnt 1) has higher Ca^2+^ affinity as compared with other subunits in peripheral tissues [33]. Schevzov *et al.*, (2005)[34] reported that the specific features of neuronal morphogenesis during embryonic stage were regulated by the troponin complex. Tnnt 1 gene function has mechanistic associations to calcium homeostasis, which in turn is associated with neurogenesis, and neuronal migration [24].

Using RNA *in situ* hybridizations, we confirmed that RNA expression of Tnnt 1 was induced in mouse brain tissue after exposures to ketamine, ionizing radiation and IFN-α [32]. We speculate that the expression of Tnnt 1, a gene associated with calcium homeostasis, in the CNS may be a common and early molecular stress-response and that the mechanism of Tnnt 1 as a CNS molecular stress biomarker may be associated with disturbance of calcium homeostasis and neurogenesis and neuronal migration**Fig. (**6**). We believe that the induced expression of Tnnt 1 and co-modulated biomarkers of calcium signaling/other neuronal pathways in adult CNS will serve as a sensitive early indicator of CNS molecular stress that is predictive of neuropathological changes in the hippocampal zone of neurogenesis and the amygdala. Future studies to investigate the temporal expression relationship between the Tnnt 1 and calcium signaling markers and the temporal expression relationship between the calcium signaling pathway and neurogenesis are warranted Fig. (**6**).

### A Comprehensive Panel of Transcriptional Biomarkers will be Needed to Measure and Monitor CNS Stress

It is naïve to expect that any single gatekeeper function and biomarker will be sufficiently robust to identify all CNS stress mechanisms underlying neuropsychiatric diseases. A growing body of evidence suggests that neuropsychiatric disorders are the consequences of dysregulation of complex intracellular signaling cascades and neuronal networks, rather than the consequences of deficits or excesses of individual neurotransmitters [35]. To capture the complexity of CNS stress response at a more global or “-omics” level, we examined gene expression profiles to identify a panel of CNS stress biomarkers and stress responses mechanisms associated with several different types of cognitive dysfunction. 

As an example of our *in vivo* systems approach, we compared the CNS transcriptional profiles of mice exposed to the stress exposure of low-dose ionizing radiation versus published CNS signatures associated with normal aging and Alzheimer’s disease. Specifically, we compared our set of radiation response genes against a compendium of data sets of transcriptional profiles of various genetic and disease conditions including those from individuals with diminished cognitive function (L2L microarray analysis tool, version 2007.1[23]) to identify shared pathways and functions. Two knowledge bases shared significant overlap with our set of down regulated genes of mice exposed to low dose radiation: the aging human brain [36] and patients with Alzheimer’s disease [37]. IPA was applied to the set of genes that was modulated in postmortem human cortex tissue from individuals of advanced ages up to 106 years old [n = 30][36], and to the gene set that was modulated in hippocampal tissue from Alzheimer’s patients [n = 22][37]. Comparison of the relative pathway enrichments for low-dose radiation mouse, human aging and Alzheimer’s disease gene expression profiles showed remarkably consistent findings not only in the direction of expression modulation (predominantly down-regulation) but also in the specific pathways involved (Table **2**). 

Six pathways that are known to be associated with neuropsychiatric diseases were in full agreement among all data sets. Five out of six showed consistent down-regulation (Table **2**) and the integrin signaling pathway was the only pathway that was consistently up-regulated. In addition, three pathways (axonal guidance signaling, actin cytoskeleton signaling, and ERK/MAPK signaling) were significantly affected in all three data sets, but they differed somewhat in the direction of their effects (up- or downregulation)[28].

Our findings suggest that low-dose irradiation modulates the expression of gene pathways that are also involved in human cognitive dysfunction associated with normal aging and Alzheimer’s disease. These findings support our hypothesis that there may be common mechanisms of CNS stress response, independent of the nature of the stressor. Future studies are warranted to determine whether other CNS stressors show similar responses and to characterize the time course of induction, duration, and persistence of stress responses in relation to the onset of neuropsychiatric illnesses.

## CONCLUDING REMARKS AND CLINICAL IMPLICATIONS

We demonstrated that the expression of Tnnt 1 was significantly induced in mouse brain tissue, including in the choroid plexus, after *in vivo* exposures to ketamine, ionizing radiation and IFN-α, all of which are known to increase the risks of neuropsychiatric diseases. The involvement of the choroid plexus which produces CSF raising the interesting possibility that CNS stress biomarkers may be detected in CSF in the future. Using a systems biology approach, we demonstrated that six neural signaling pathways associated with neuropsychiatric diseases were concordantly modulated in mouse brain tissue after low-dose (10 cGy) irradiation, in normal aging of the human brain (unirradiated), and in brain tissue from patients with Alzheimer’s disease. This suggests that diverse CNS stressors and pathologies can share common molecular stress response mechanisms.

Our systems approach addresses a critical challenge of how to identify candidate biomarkers of early CNS stress for validation studies towards applications for preclinical diagnoses and for monitoring the treatments of neuropsychiatric diseases. Effective biomarkers of the CNS stress responses will open doors to urgently needed advances in the development and testing of new diagnostic and treatment strategies for neuropsychiatric disorders. Our studies provide several specific insights into the mechanisms of CNS stress-responses that may become useful for developing early diagnostics for CNS stress (as examples: (1) the association with Calcium signaling may lead to the development of brain imaging methods base on calcium ligands, and (2) choroid plexus secretions associated with stress responses may be detectable in the CSF as protein biomarkers or as other molecular signatures). CNS stress response biomarkers and molecular stress signatures hold the promise to enable rapid advances in early diagnosis and, hopefully, to provide mechanism-based treatments options of neuropsychiatric diseases in the future. 

## SUPPLEMENTARY MATERIAL

Supplementary material is available on the publishers Web site along with the published article.

## Figures and Tables

**Fig. (1). Working hypothesis of the link between CNS stress and risks of neuropsychiatric diseases.**
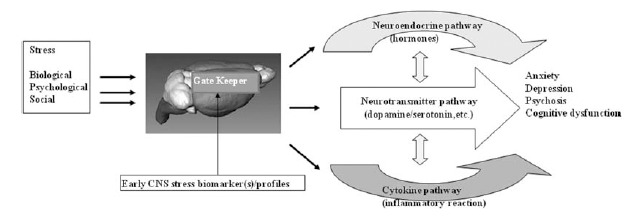
We hypothesize that there are common early stress response mechanisms (gate keepers) in the CNS that are induced after exposures to a broad variety of environmental stress/insults, and that these mechanisms are associated with increased risks for a broad range of neuropsychiatric diseases.

**Fig. (2). Experimental designs.**
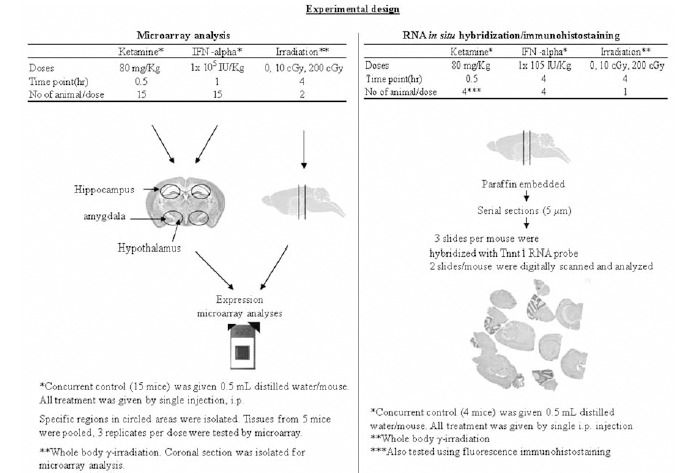
Left panel: Microarray analyses. Right panel: RNA *in situ* hybridization and fluorescence immunohistostaining.

**Fig. (3). Gene Ontology (GO) analyses: Biological Process.**
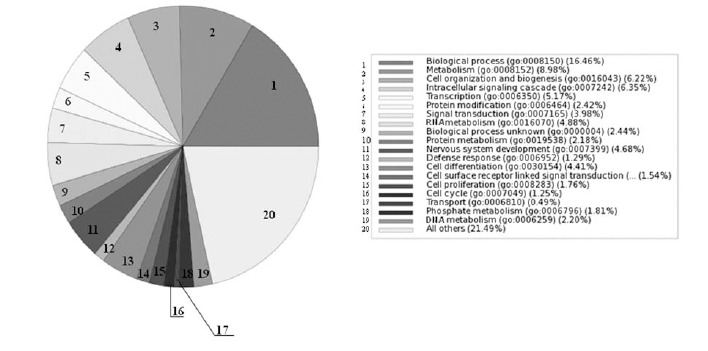
GO analysis of biological processes associated with differentially expressed genes in brain tissue of mice treated with ketamine.

**Fig. (4). Tnnt 1 gene interaction network.**
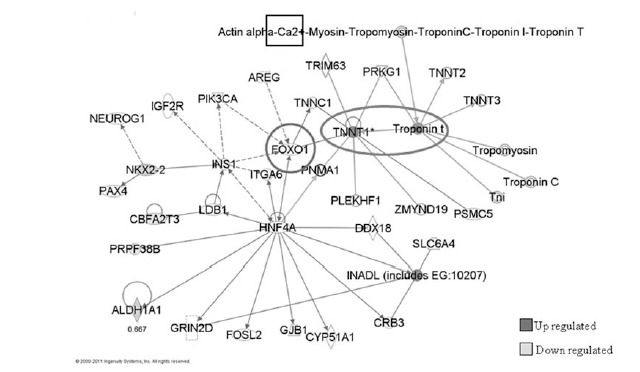
IPA network analyses were applied to differentially expressed genes in mouse brain after in vivo ketamine exposure. The two top networks were merged to create the gene interaction network that involved Tnnt 1 (oval), which is regulated by Fox O1 (circle) and involved in the Calcium signaling pathway (rectangle). Dark gray color: up-regulated genes. Light gray color: down-regulated genes. White color: genes that were not differentially expressed after ketamine treatment.

**Fig. (5). Comparison of protein and RNA expression of Tnnt1 in brain tissue of mice treated with ketamine.**
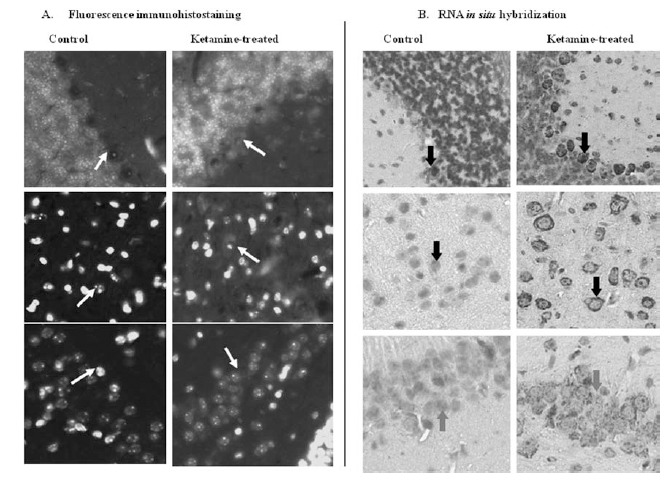
(**A**) Fluorescence immunohistostaining. Fluorescence red cytoplasm staining indicated Tnnt 1 protein expression. (**B**) RNA *in situ* hybridization. Dark gray cytoplasm staining indicated Tnnt 1 RNA expression. Top panels: Purkinje cells of cerebellum; middle panels: pyramidal cells; bottom panels: Hippocampus. The indication of arrows: Top panels: Purkinje cells; middle panels: pyramidal cells; bottom panels: hippocampus. (Magnification: 40X). (Reprinted from NeuroToxicology 30 (2009) 261–268, Lowe *et al*., Fig. (**4**)[32], with permission from Elsevier).

**Fig. (6):**
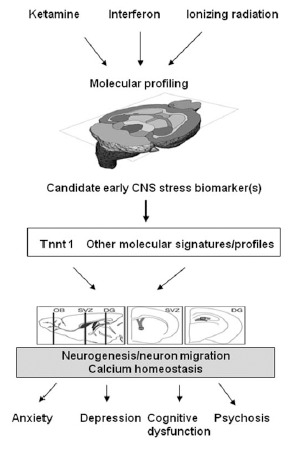
*In vivo* system approach to validating candidate biomarkers of early CNS stress.

**Table 1.** Comparisons of RNA Expression Patterns of Tnnt 1 in Adult Brain Tissue of Mice that Received *in vivo* Exposures of Three CNS Stress Agents*

	Controls (Concurrent)§	Ketamine	Radiation***	Interferon-α
Dose	n/a	80 mg/Kg	10 cGy, 200 cGy	1x 10^5^ IU/Kg
Sampling Time Post Treatment (Hour)	Concurrent	0.5	4	4
**Exposure-induced Expression Patterns in:**				
Choroid Plexus	Positive	Enhanced	Enhanced	Enhanced
Ependymal Lining of Ventricles	Positive	Enhanced	Enhanced	Enhanced
Hippocampus				
Ammon's Horn (CA1 - CA3 Regions)	Positive	Enhanced	Enhanced	Enhanced
Dentate Gyrus Region	Negative	Induced	Induced	Induced
Ammon’s Horn (A) vs Dentate Gyrus Region (DG)	A > DG	A > DG	A = DG	A > DG
Purkinje Cells of Cerebellum	Negative	Induced**	Induced	Not Induced
Pyramidal Neurons of Cereberal Cortex	Negative	Induced	Induced	Induced
Amygdala	Negative	Induced	Induced	Induced
Glial Cells	Negative	Not Induced	Not Induced	Not Induced

§ Concurrent controls for ketamine and interferon received i.p. injection with distilled water; radiation controls were sham exposed.

Concurrent controls for all three stressors showed similar expression patterns. n/a: not applicable; positive: expression detectable in unexposed; negative: not detectable

* Implication of RNA expression patterns of Tnnt 1 change: from weak to strong as "enhanced"; from negative to positive as "induced".

** About 200 Purkinje cells per animal were scored, cells with Tnnt 1 stain scored as induced.

Comparison was made between the ketamine-exposed and control animals, p < 0.0001 [31]

*** Similar response in 10 cGy and 200 cGy animals, compared to control.

Modified from NeuroToxicology 30 (2009) 261–268, Lowe *et al*., Table 1, [32].

**Table 2.** Knowledgebase Comparisons Identified Several Low-Dose Cognition-Related CNS Pathways that are Similarly Affected in the Aging Human Brain and in the Brain of Alzheimer’s Disease Patients

Pathway	Mouse	Human	
	Low-Dose Irradiation Brain*	Aging**	Alzheimer's Disease***
**Integrin Signaling**	⇑	⇑	⇑
**Synaptic Long Term Depression**	⇓	⇓	⇓
**Synaptic Long Term Potentiation**	⇓	⇓	⇓
**cAMP Mediated Signaling**	⇓	⇓	⇓
**G-Protein Coupled Receptor Signaling**	⇓	⇓	⇓
**Glutamate Receptor Signaling**	⇓	⇓	⇓
**Actin Cytoskeleton Signaling**	⇔	⇔	⇔
**Axonal Guidance Signaling**	⇔	⇔	⇔
**ERK/MAPK Signaling**	⇔	⇔	⇔

* Brain tissue from mice that received 10 cGy of whole-body radiation (unique low-dose genes)[28].

** Brain tissue from aging human [36].

*** Hippocampal brain tissue from Alzheimer’s disease patients [37].

⇑ Component genes of pathway were generally upregulated.

⇓ Component genes of pathway were generally down regulated.

⇔ Component genes of pathway modulated in variable directions.
