# Supplementary material for: Characterization of the Early CNS Stress Biomarkers and Profiles Associated with Neuropsychiatric Diseases
Source: Curr Genomics. 2012 Sep;13(6):489–97. doi: 10.2174/138920212802510448 (PMC3426783; doi:10.2174/138920212802510448)
Supplement: Supplementary file 1 [file CG-6-489_SD1.pdf]

SUPPLEMENTARY MATERIAL

Table S1. Up Load, Microarray Data Collected from Mice Treated with Ketamine (By Single i.p. Injection at 80 mg/kg, and Brain Tissue was Isolated at 30 Mins Post-Treatment); Or With IFN-a (By a Single i.p. Injection at 1 x 10^5 IU/kg, and Brain Tissue was Isolated At One Hour Post Treatment)

Control1	Control2	Control3	Interferon1	Interferon2	Interferon3	Ketamine1	Ketamine2	Ketamine3	M	t	P.Value	B	Acontrolcon-trolNUM	SYMBOL	NAME
4.793354	4.654879	4.698088	4.847143	4.855053	4.741917	6.651852	6.432063	6.623879	1.8538246	24.850029	9.79E-06	8.6932531	AF060539	Cipp	Channel-interacting PDZ domain protein
9.057001	9.157884	8.863017	8.934456	8.789569	8.874863	10.541494	10.349273	10.353497	1.388787	15.599836	3.40E-04	7.0416587	AB011812	Prkcd	Protein kinase C, delta
7.577778	7.73207	7.350905	7.424783	7.421164	7.437583	10.02065	9.799643	9.341178	2.1669061	13.789938	6.85E-04	6.4460635	X60304	Prkcd	Protein kinase C, delta
6.393417	6.457301	6.48245	6.234397	6.333516	6.305602	7.143646	7.085424	7.151725	0.6825419	12.279049	1.35E-03	5.8273314	AV349152	Rgs16	Regulator of G-protein signaling 16
8.399448	8.539985	8.510253	8.552129	8.575991	8.461284	10.22439	10.217665	10.917404	1.9699248	12.061467	1.35E-03	5.7272787	AV213431	Tnnt1	Troponin T1, skeletal, slow
5.523297	5.680001	5.522982	5.666089	5.622153	5.403955	6.397872	6.511414	6.600763	0.9279229	10.573343	3.55E-03	4.9550397	Z48800	Gbx2	Gastrulation brain homeobox 2
8.935453	8.745386	8.970718	8.475101	8.340794	8.418088	7.974821	8.270076	7.92832	-0.8261134	-8.303827	2.37E-02	3.4079852	AF039391	Crym	Crystallin, mu
9.105795	8.998918	9.131153	9.435459	9.527771	9.71017	8.450374	8.200586	8.408407	-0.7254998	-7.968258	2.91E-02	3.1321248	M24377	Egr2	Early growth response 2
6.827152	6.80744	6.641851	6.790951	6.706873	6.704604	7.368487	7.461598	7.768459	0.7740334	7.617354	3.54E-02	2.8285331	AJ131711	Tnnt1	Troponin T1, skeletal, slow
12.354706	12.438261	12.493957	12.467447	12.489629	12.500242	12.825943	12.849004	12.83726	0.4084276	7.470183	3.54E-02	2.6964036	AI839662	Mobp	Myelin-associated oligodendrocytic basic protein
10.917203	11.074564	11.033938	10.932048	10.891264	11.034077	10.584088	10.43585	10.419302	-0.5288217	-7.379515	3.54E-02	2.6135475	AJ002387	Hspa5	Heat shock 70kD protein 5 (glucose-regulated protein)
10.267126	10.248328	10.230788	10.337442	10.509457	10.322842	10.714752	10.995343	11.036427	0.6667599	7.361871	3.54E-02	2.5972936	M74570	Aldh1a1	Aldehyde dehydro-genase family 1, subfamily A1
11.231517	11.152513	11.168858	11.283131	11.364768	11.488894	10.75337	10.545545	10.591108	-0.5542886	-7.328611	3.54E-02	2.5665369	M28845	Egr1	Early growth response 1
9.236996	9.393145	9.275561	9.144793	9.091416	9.352289	9.802483	9.892261	9.897223	0.5620882	7.081956	4.33E-02	2.3336321	M35131	Nefh	Neurofilament, heavy polypeptide
6.63872	6.874939	6.859769	6.736948	6.863286	6.696909	7.327857	7.424488	7.630502	0.6698067	6.972321	4.36E-02	2.2273339	Y18723	Prss18	Protease, serine, 18
7.471764	7.434935	7.333119	7.320656	7.23711	7.43902	6.934293	6.89314	6.695767	-0.5722066	-6.956745	4.36E-02	2.2120911	AI846392	Nsap1-pending	NS1-associated protein 1
7.023881	7.145065	6.938649	6.979177	7.063584	7.078587	7.516016	7.931356	7.782261	0.707346	6.898197	4.39E-02	2.154479	X13986	Spp1	Secreted phos-phoprotein 1
8.51296	8.458285	8.253437	8.27619	8.182608	8.238235	9.478658	9.143412	9.000627	0.7993386	6.79414	4.47E-02	2.050842	U94828	Rgs16	Regulator of G-protein signaling 16
9.204243	9.341841	9.306217	9.165789	9.193405	9.298367	9.713949	9.666796	9.783578	0.437341	6.787716	4.47E-02	2.0443918	AW124433	Adarb1	Adenosine deaminase, RNA-specific, B1
4.506037	4.496852	4.499559	4.509611	4.573572	4.597821	4.833513	4.815597	4.889315	0.3453257	6.598234	5.08E-02	1.8513423	AA762212	Serpinb1a	Serine (or cysteine) proteinase inhibi-tor, clade B, member 1a
7.505272	7.672157	7.6642	7.6243	7.725999	7.35228	8.177169	8.318286	8.286115	0.6466468	6.592272	5.08E-02	1.8451805	L08074	Nkx6-2	NK6 transcription factor related, locus 2 (Drosophila)
5.602471	5.849492	5.947231	5.668012	5.647317	5.652464	5.126685	5.045063	5.317775	-0.6365568	-6.50813	5.14E-02	1.7576307	AF075136	Sap30	Sin3 associated polypeptide
5.332276	5.506446	5.838403	5.445402	5.267807	5.213732	7.839481	7.693346	6.609645	1.8217827	6.506442	5.14E-02	1.7558633	U66918	Shox2	Short stature homeobox 2
6.661341	6.781542	6.794822	6.542224	6.644018	6.667639	6.362451	6.351259	6.357806	-0.3887303	-6.442077	5.32E-02	1.6881398	AV374868	Socs3	Suppressor of cytokine signaling

Control1	Control2	Control3	Interferon1	Interferon2	Interferon3	Ketamine1	Ketamine2	Ketamine3	M	t	P.Value	B	Acontrolcon-trolNUM	SYMBOL	NAME
															3
6.721865	6.852476	6.833941	6.754992	6.849755	6.605311	7.276517	7.364924	7.605005	0.6127214	6.370285	5.32E-02	1.6118416	U31566	Nkx2-2	NK2 transcription factor related, locus 2 (Drosophila)
8.849966	10.279462	8.763038	9.512223	8.611027	9.488916	7.08323	6.743585	6.630215	-2.4784783	-6.362706	5.32E-02	1.6037399	AF109906	Bat8	HLA-B associated transcript 8
7.129764	7.227618	7.401071	6.937637	7.008073	7.154188	6.759791	6.486166	6.350288	-0.7207358	-6.345195	5.32E-02	1.5849865	AF038029	Cbfa2t3h	Core-binding factor, runt domain, alpha subunit 2; translocated to, 3 homolog (human)
8.670098	8.43206	8.616377	7.701789	7.920789	7.918332	7.471174	8.024623	7.400248	-0.9408298	-6.144163	6.56E-02	1.3662222	X53929	Dcn	Decorin
7.123789	7.019467	7.049004	7.042333	6.884075	6.963148	7.49338	7.430006	7.39447	0.3751989	6.01075	7.28E-02	1.2174711	M32502	Wnt3	Wingless-related MMTV integration site 3
6.199566	6.275429	6.29345	6.238118	6.186998	6.340904	5.80456	5.929233	5.889142	-0.3818367	-6.006236	7.28E-02	1.2123869	AW125643	NA	NA
9.408189	9.583731	9.758641	9.293963	9.211877	9.48728	9.025055	8.879772	9.019641	-0.6086975	-5.95327	7.53E-02	1.1524922	AW049897	Fln29-pending	FLN29 gene product
5.723756	5.807034	5.974427	5.497695	5.626548	5.535769	5.4438	5.302151	5.393972	-0.455098	-5.9118	7.61E-02	1.1052774	Z48587	Rala	v-ral simian leukemia viral oncogene homolog A (ras related)
8.970167	9.034941	8.891057	9.031776	8.73564	8.889872	9.469547	9.381842	9.582328	0.5125172	5.896119	7.61E-02	1.0873496	M34896	Evi2	Ecotropic viral integration site 2
8.717788	8.647051	8.787662	8.479931	8.480335	8.563318	8.203023	8.171003	8.424938	-0.4511792	-5.82365	7.96E-02	1.0039765	AJ223362	Myh7	Myosin, heavy polypeptide 7, cardiac muscle, beta
5.672201	5.369792	5.534566	5.416555	5.673859	5.678624	5.046059	4.896534	4.920168	-0.571266	-5.814973	7.96E-02	0.9939354	M88242	Ptgs2	Prostaglandin-endoperoxide synthase 2
8.814494	8.612345	8.89685	8.344897	8.623131	8.656166	8.251621	8.195412	8.160912	-0.5719145	-5.783518	8.06E-02	0.9574325	AW047710	Zfp312	Zinc finger protein 312
9.925206	9.650359	9.696719	9.419058	9.419456	9.679278	9.209076	9.27293	9.161513	-0.5429216	-5.723272	8.47E-02	0.8870594	AI853930	2810012H18Rik	RIKEN cDNA 2810012H18 gene
9.666045	10.93212	9.756657	10.030016	9.736049	10.012575	8.563834	8.56564	8.444016	-1.5937776	-5.698963	8.48E-02	0.8584932	M12571	Hspa1a	Heat shock protein 1A
10.047879	9.857237	9.753324	10.256112	10.156492	10.337502	9.387162	9.132951	9.421084	-0.5724145	-5.682466	8.48E-02	0.83905	M38381	Clk	CDC-like kinase
8.442008	8.478237	8.3531	8.279963	8.36945	8.264795	8.016346	7.882001	7.531582	-0.6144718	-5.553191	9.49E-02	0.6851171	AF055638	Gadd45g	Growth arrest and DNA-damage-inducible 45 gamma
10.880742	10.831329	10.820376	10.809124	10.829748	10.946019	11.19976	11.146449	11.142498	0.3187537	5.547795	9.49E-02	0.6786311	X61452	4-Sep	Septin 4
4.552205	4.63447	4.779357	4.619975	4.49401	4.569637	4.279243	4.248569	4.290951	-0.3824235	-5.540565	9.49E-02	0.6699326	AW107922	Sox11	SRY-box containing gene 11
8.18813	8.044699	7.853102	8.065379	7.900723	7.909443	7.514587	7.505206	7.161549	-0.634863	-5.50656	9.62E-02	0.6289024	AI837467	1110021E09Rik	RIKEN cDNA 1110021E09 gene
6.437284	6.202431	6.382599	6.010553	6.027076	6.166125	5.935358	5.816762	5.96333	-0.4356218	-5.488219	9.62E-02	0.6066907	Y14334	Alox12b	Arachidonate 12-lipoxygenase, 12R type
6.910279	6.887138	7.063001	6.869153	6.972305	7.125267	6.513663	6.569596	6.496008	-0.4270502	-5.457995	9.62E-02	0.569965	U83148	Nfil3	Nuclear factor, interleukin 3, regulated
10.051926	10.004714	10.060222	9.56739	9.791002	9.558996	9.577229	9.719408	9.536297	-0.4279758	-5.454714	9.62E-02	0.5659689	AW124186	Smarca4	SWI/SNF related, matrix associated, actin dependent regulator of chromatin, subfamily a, member 4
9.543962	9.274727	9.583039	9.404581	9.302149	9.767588	8.838313	8.667977	8.824295	-0.6903813	-5.440704	9.62E-02	0.5488859	X14897	Fosb	FBJ osteosarcoma oncogene B
9.976571	9.90276	10.012389	9.775571	9.888287	9.808341	9.695723	9.600135	9.499795	-0.3653559	-5.428719	9.62E-02	0.5342441	AI852165	D10Jhu81e	DNA segment, Chr 10, Johns Hopkins

Control1	Control2	Control3	Interferon1	Interferon2	Interferon3	Ketamine1	Ketamine2	Ketamine3	M	t	P.Value	B	Acontrolcon-trolNUM	SYMBOL	NAME
															University 81 expressed
5.844026	5.700688	5.808726	5.720032	5.732904	5.715826	5.434147	5.521234	5.334227	-0.3546106	-5.414053	9.62E-02	0.5162947	U56649	Pde1a	Phosphodiesterase 1A, calmodulin-dependent
7.224216	7.148074	7.198389	6.922666	7.094441	7.060114	6.819049	6.850297	6.89953	-0.3339346	-5.374898	9.94E-02	0.468196	AA968123	Nav1	Neuron navigator 1

SUPPLEMENTARY FIGURES

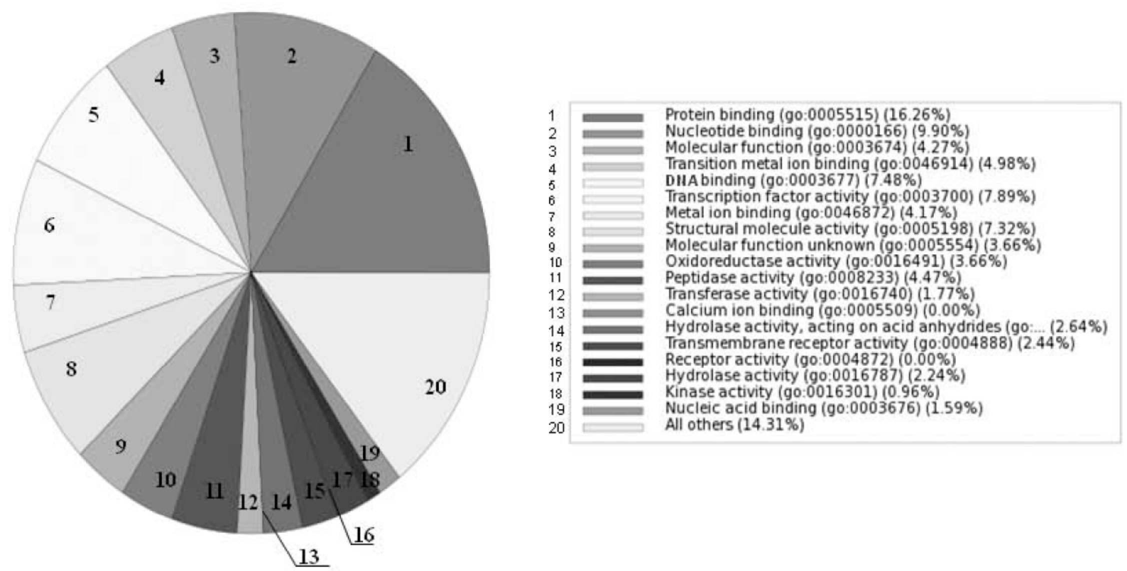

(Fig. S1)

Figure S1.

Title: Gene Ontology (GO) analyses: Molecular Function.

Legend: GO analyses of molecular function with modulated signatures in mouse brain induced by ketamine treatment.

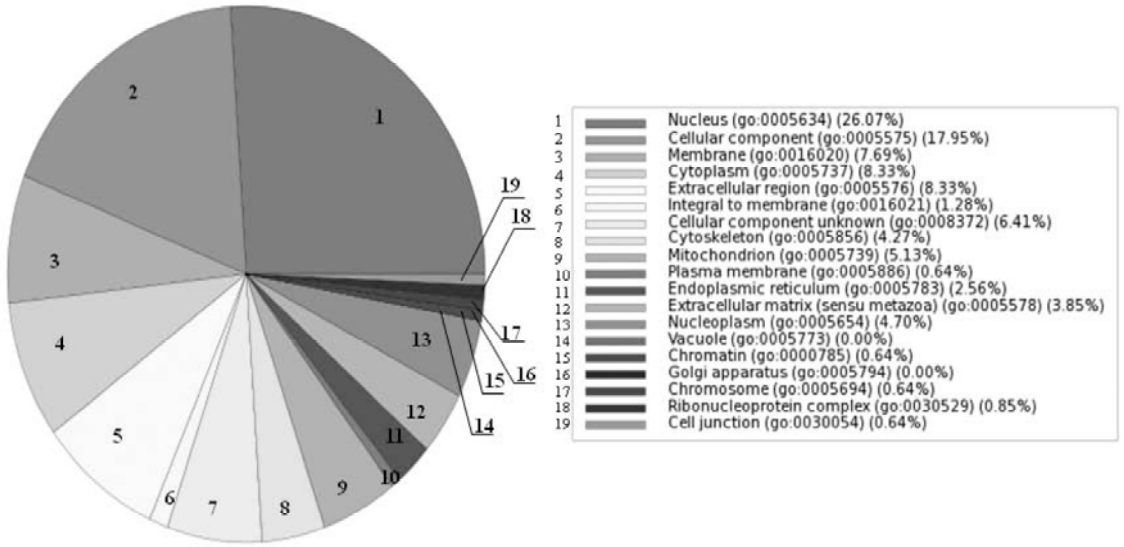

(Fig. S2)

Figure S2.

Title: Gene Ontology (GO) analyses: Cellular Component.

Legend: GO analyses of cellular component with modulated signatures in mouse brain induced by ketamine treatment.
